# Incidence and risk factors of preoperative isolated calf deep venous thrombosis following hip fractures

**DOI:** 10.1097/MD.0000000000029140

**Published:** 2022-03-25

**Authors:** Weiguang Zhao, Jianlong Zhao, Tiantian Liu, Zhenwu Liu, Li Liu

**Affiliations:** a *Department of Orthopaedic Surgery, Handan Central Hospital, Handan, Hebei, People's Republic of China,*; b *Graduate School of Hebei North University, Zhangjiakou, Hebei, People's Republic of China,*; c *Chengde Medical University, Chengde, Hebei, People's Republic of China.*

**Keywords:** epidemiology, hip fractures, isolated calf deep venous thrombosis, risk factors

## Abstract

There is still a lack of data on isolated calf deep vein thrombosis (ICDVT) following hip fractures surgery. The study aimed to determine the incidence of preoperative ICDVT and the associated risk factors in patients with hip fractures requiring surgery.

The 289 patients who required hip surgery were included, duplex ultrasonography was routinely used to make a definite diagnosis of preoperative ICDVT located in unilateral or bilateral calf. Data on demographics and laboratory-associated blood biomarkers results were included. Univariate analyses were used to analyse the data of demographics, comorbidities, personal history operation related indexes and laboratory biomarkers, then the multivariate logistic regression analysis was employed to identify the independent risk factors associated with ICDVT.

Sixty-eight (23.5%) patients were diagnosed with preoperative ICDVTs. The univariate analyses showed significant differences regarding ICDVT were age, current smoking, alcohol consumption, time from injury to operation, albumin, white blood cells, lymphocyte, red blood cells, hemoglobin, hematocrit, and activated partial thromboplastin time level among the 44 factors. The multivariable model confirmed 3 risk factors were significantly independent in association with preoperative ICDVTs, including current smoking, time delay from injury to operation and activated partial thromboplastin time ( < 28seconds).

The incidence of preoperative ICDVT in hip fracture was 23.5%, and patients with associated risk factors are prone to form ICDVTs, identification of these factors may help to reduce the incidence of ICDVT with hip fractures by taking early prevention measures.

## 1. Introduction

Isolated calf deep venous thrombosis (ICDVT) includes thrombosis which is located at the far end of the popliteal vein, such as an anterior tibial vein, posterior tibial vein, fibular vein and the intramuscular vein of soleus and gastrocnemius. This type of thrombosis has the highest incidence, about half of all deep vein thrombosis (DVT). In recent years, more and more evidence has shown that ICDVT can develop into proximal DVT, even cause pulmonary embolism (PE). Robert-Ebadi and Righini^[1]^ reported that ICDVT diagnosed by intravenous ultrasound accounted for 30% to 50% of all lower extremity DVTs, and the proportion of outpatients was even higher, about 60% to 70%. In addition, as the global population ages, the incidence of hip fractures is gradually increasing, which has become an increasingly serious worldwide public health problem. It is estimated that by 2050, the number of hip fractures worldwide will reach more than 6 million, and the majority of them are the aged, who also belong to the high-risk group of ICDVT.^[2]^ Most hip fractures require surgical treatment, and satisfactory results can be achieved through surgical treatment.^[3]^ However, preoperative ICDVT may lead to delayed surgery in such patients, which would significantly increases postoperative mortality and morbidity, and adversely affects quality of life.^[4]^ If not given enough attention to the progression of ICDVT in such a population usually cause a poor prognosis, while if we first identify the risk factors associated with preoperative ICDVT, then simple early interventions may obtain satisfactory results. Studies^[2,5-8]^ had shown that the incidence of DVT after hip fractures was the highest, approximately 12.9% to 30.0%, and the incidence of ICDVT was approximately 8 times that of proximal DVT. Therefore, it is necessary to study the occurrence of preoperative ICDVT and related risk factors of this type of fracture. So we conducted this retrospective study aimed to clarify: the incidence of preoperative ICDVT following hip fractures surgery; and whether some risk factors, including hematological parameters that are easily available clinically, demographics, comorbidities, personal history and so on, were independently associated with increased risk of preoperative ICDVT in hip fractures. This study might help orthopaedic surgeons in performing ICDVT risk stratification, cross-speciality decision making and targeted precaution measures to reduce the incidence of early and late complications such as proximal DVT, PE, and post-thrombotic syndrome, decrease the mortality and subsequent medical costs, and improve the patient's quality of life.

## 2. Materials and methods

### 2.1. Patients

This retrospective study was approved by the ethics committee of Handan Central Hospital of Hebei province and the informed consent was written by all the participants. All the patients included in this study were diagnosed with hip fractures (including femoral neck, intertrochanteric and subtrochanteric fractures) and underwent surgery in Handan Central Hospital between February 2015 and December 2020. Blood tests from hospitalization to preoperation, regular duplex ultrasonography (DUS) screening and thromboprophylaxis therapy were con-ducted according to our institutional protocol. All the baseline characteristics, blood test parameters and ICDVT results were extracted from the electronic medical record system. Inclusion criteria were as follows:

1. Patients aged 18 years or older;
2. Definite diagnosis of hip fracture and be treated surgically;
3. Complete medical records and DUS, blood test results available.

Exclusion criteria were:

1. Open fractures;
2. Pathological or old fractures;
3. Polytrauma or concurrent fracture in other limbs;
4. History of venous thromboembolism;
5. Recent use of anticoagulants or oral contraceptives within 3 months for other illnesses;
6. Incomplete data;
7. DVT located in popliteal vein and above.

### 2.2. ICDVT detection and prophylaxis

Patients received DUS on bilateral lower extremities the same day at admission, subsequently once per 2 days and the day before the operation, or when any signs and symptoms suggestive of ICDVT presented. ICDVT was diagnosed by DUS following the guideline for diagnosis and treatment of DVT updated by the Chinese Orthopaedic Association and Chinese Medical Association.^[9]^ ICDVT was confirmed based on the detection of venous lumen obstruction or filling defect in posterior tibial vein, anterior tibial vein, peroneal vein, soleal vein and gastrocnemius vein. Thromboprophylaxis regimen was performed for each patient from hospitalization to preoperation, consisting of elastic compression stockings, intermittent pneumonic compression, and chemoprophylaxis. Prophylactic low-molecular-weight heparin (4100 IU, once daily) was given within the first 24 hours after admission and withheld 12 hours before surgery. The elastic compression stockings and intermittent pneumonic compression were stopped once ICDVT was diagnosed.

### 2.3. Data collection

DUS and blood test results were collected. The demographic data, consisting of gender, age, body mass index, fractures of limbs, current smoking, alcohol consumption, time from injury to operation, American Society of Anesthesiologists classification was extracted from the electronic medical record system. The comorbidities included diabetes mellitus, hypertension and heart disease were also inquired from the medical record. Overnight fasting blood samples were drawn for test preoperation. Data were extracted from the results that were closest to the time of ICDVT diagnosis if multiple blood tests were performed. Hematological biomarkers included alkaline phosphatase, albumin level, globulin level, alanine transaminase, aspartate transaminase, fasting blood glucose, total bilirubin, direct bilirubin, indirect bilirubin, gamma-glutamyl transpeptidase, uric acid, cholinesterase, hypersensitive C-reactive protein, high-density lipoprotein cholesterol level, low-density lipoprotein cholesterol level, serum sodium concentration, serum potassium concentration, serum chloride concentration, white blood cell count, neutrophil count, lymphocyte count, monocyte, red blood cell count, hemoglobin level, hematocrit, platelet, platelet distribution width, total cholesterol level, triglyceride level, thrombin time, activated partial thromboplastin time, thrombin time, and fibrinogen level.

### 2.4. Statistical analysis

SPSS 25.0 (IBM, Armonk, NY) was used to perform all the statistical analyses. Continuous data corresponding to a normal distribution was presented by the Student *t* test, and those that did not coincide with normal distribution were evaluated by Mann-Whitney *U* test, described as mean±standard deviation/median with quartile. Categorical data were evaluated by chi-square or Fisher exact test, as appropriate, expressed as number and percentage (%). All the categorical variables with *P* < .10 from the results of univariate analyses entered the multivariate logistics regression model to identify the independent predictors of ICDVT, and the correlation strength was indicated by odds ratio (OR) and 95% confidence interval. *P* values < .05 were regarded as statistically significant. Hosmer-Lemeshow test was applied to assess the fitness of the final model, and *P* > .05 was considered to be an acceptable result.

## 3. Results

We initially included 326 patients, after screening by exclusion criteria, in our cohort, the analyses of overall 289 patients with surgically treated hip fractures were conducted, comprising 109 males and 180 females, 68 cases developed a preoperative ICDVT, indicating a morbidity of 23.5%. Among the ICDVTs, the proportion of female patients was higher than that of male patients, the former accounted for 64.7%. However, there was no significant difference in gender composition between ICDVT group and non-ICDVT group (*P* = .637) (Table 1). But the patients in ICDVT group was older than control group (74.01± 12.4 vs 68.98±16.1, *P* = .007) (see Table 1). Except for age, univariate analyses also showed significant differences regarding DVT were current smoking (*P* = .001), alcohol consumption (*P* = .003), time from injury to operation (*P* = .019), albumin (*P* = .003), white blood cells (*P* = .016), lymphocyte (*P* = .030), red blood cells (*P* = .011), hemoglobin (*P* = .008), hematocrit (*P* = .042) and APTT (*P* = .017) among the 44 factors. The abovementioned variables were entered into the multivariable model. Finally, 3 variables demonstrated significantly independent association with preoperative ICDVTs, including current smoking (OR = 3.053, *P* = .003), time from injury to operation (OR = 1.076, *P* = .032) and APTT (OR = 4.772, *P* = .025) (Table 2). The Homser-Lemeshow test showed good fitness of the final model (X^2^ = 6.275, *P* = .616).

**Table 1:** Univariate analyses of risk factors associated with preoperative ICDVT following hip fractures.

**Variables**	**No. (%) of ICDVT (n = 68)**	**No. (%) of non-ICDVT (n = 221)**	***P*** **value**
Gender (male)	24 (35.3)	85 (38.5)	.637
Age	74.01±12.4	68.98±16.1	.007
BMI	23.71±3.5	23.36±2.5	.365
Diabetes mellitus	11 (16.2)	31 (14.0)	.660
Hypertension	16(23.5)	60 (27.1)	.553
Heart disease	7 (10.3)	19 (8.6)	.669
Fractures of limbs (left)	38 (55.9)	106 (48.0)	.253
Current smoking	18 (26.5)	22 (10.0)	.001
Alcohol consumption	13 (19.1)	15 (6.8)	.003
Time from injury to operation, d	7.05±4.3	5.76±3.8	.019
ASA (III and above)	42 (61.8)	110(49.8)	.083
ALP( > 125U/L)	4 (5.9)	17 (7.7)	.814
ALB ( < 35 g/L)	27 (39.7)	48 (21.7)	.003
GLOB( < 20 g/L)	6 (8.8)	8 (3.6)	.154
ALT( > 40 U/L)	7 (10.3)	12 (5.4)	.256
AST( > 35 U/L)	8 (11.8)	24 (10.9)	.835
FBG ( > 6.1 mmol/L)	33 (48.5)	107 (48.4)	.987
TBIL ( > 21 μmol/L)	17 (25.0)	72 (32.6)	.236
DBIL ( > 6 μmol/L)	13 (19.1)	46 (20.8)	.761
IBIL( > 14 μmol/L)	24(35.3)	87 (39.4)	.546
GGT ( > 60 U/L)	1 (1.5)	14 (6.3)	.205
UA( > upper limit)	8 (11.8)	16 (7.2)	.237
CHE ( < 5 kU/L)	14 (20.6)	41 (18.6)	.708
HCRP ( > 8 mg/L)	58 (85.3)	185 (83.7)	.755
HDL-C ( < 1.1 mmol/L)	30 (44.1)	87 (39.4)	.485
LDL-C ( > 3.37 mmol/L)	10 (14.7)	34 (15.4)	.892
Na^+^ ( < 135 mmol/L)	12 (17.6)	37 (16.7)	.862
K^+^ ( < 3.5 mmol/L)	12 (17.6)	38 (71.2)	.931
Cl^-^ ( < 99 mmol/L)	7 (10.3)	27 (12.2)	.667
WBC ( > 10 × 10^9^/L)	10 (14.7)	65 (29.4)	.016
NEU ( > 6.3 × 10^9^/L)	32 (47.1)	119 (53.8)	.327
LYM ( < 1.1 × 10^9^/L)	34 (50.0)	78 (35.3)	.030
MON ( > 0.6 × 10^9^/L)	24 (35.3)	99 (44.8)	.166
RBC < lower limit	47 (69.1)	114 (51.6)	.011
HGB < lower limit	43 (63.2)	99 (44.8)	.008
HCT < lower limit	49 (72.1)	129 (58.4)	.042
PLT ( > 350 × 10^9^/L)	4 (5.9)	8 (3.6)	.638
PDW < 12%	35 (51.5)	108 (48.9)	.707
TC ( > 5.2 mmol/L)	9 (13.2)	41 (18.6)	.311
TG ( > 1.7 mmol/L)	7 (10.3)	26 (11.8)	.739
PT ( < 9 s)	14 (20.6)	50 (22.6)	.724
APTT ( < 28 s)	6 (8.8)	4 (1.8)	.017
TT ( > 17 s)	10 (14.7)	26 (11.8)	.521
FIB ( > 4 g/L)	25 (36.8)	66 (29.9)	.284

UA, reference range: female, 155-357 μmol/L; male, 208-428 μmol/L; RBC, reference range: female, 3.8-5.1 × 10^12^/L; males, 4.3-5.8 × 10^12^/L. HGB, reference range: females, 115-150 g/L; males, 120-160 g/L; HCT, reference range: females, 35%-45%; males, 40%-50%. ALB = albumin, ALP = alkaline phosphatase, ALT = alanine transaminase, APTT = activated partial thromboplastin time, ASA = American Society of Anesthesiologists, AST = aspartate transaminase, BMI = body mass index, CHE = cholinesterase, DBIL = direct bilirubin, FBG = fasting blood glucose, FIB = fibrinogen, GGT = gamma-glutamyl transpeptidase, GLOB = globulin, HCRP = hypersensitive C-reactive protein, HCT = hematocrit, HDL-C = high-density lipoprotein cholesterol, HGB = hemoglobin, IBIL = indirect bilirubin, ICDVT = isolated calf deep vein thrombosis, LDL-C = low-density lipoprotein cholesterol, LYM = lymphocyte, MON = monocyte, NEU = neutrophil count, PDW = platelet distribution width, PLT = platelet, PT = thrombin time, RBC = red blood cell, TBIL = total bilirubin, TC = total cholesterol, TG = triglyceride, TT = thrombin time, UA = uric acid, WBC = white blood cell.

**Table 2:** Multivariate analysis of factors associated with preoperative ICDVT following hip fractures.

		**95%CI**	
**Variables**	**OR**	**Lower limit**	**Upper limit**	***P***
Current smoking	3.053	1.471	6.335	.003
Time from injury to operation, d	1.076	1.006	1.150	.032
APTT ( < 28 s)	4.772	1.214	18.752	.025
Alcohol consumption	1.467	0.320	6.726	.622
HCT	0.513	0.150	1.750	.286
HGB	1.209	0.407	3.586	.733
RBC	1.969	0.754	5.140	.166
LYM	1.512	0.830	2.754	.176
WBC	0.540	0.249	1.170	.119
ALB	1.419	0.699	2.878	.333
Age	1.020	0.999	1.042	.065

Data was presented as *P* value.

ALB = albumin, APTT = activated partial thromboplastin time, CI = confidence interval, HCT = hematocrit, HGB = hemoglobin, ICDVT = isolated calf deep vein thrombosis, LYM = lymphocyte, OR = odds ratio, RBC = red blood cell, WBC = white blood cell.

## 4. Discussion

Hip fracture is the most common in the elderly, and low energy damage can cause the fracture of the area, often require surgical treatment after fracture.^[10-13]^ That was consistent with the results of this study that the patients included were mainly elderly. Due to the characteristics of the elderly themselves, it will take a long time to improve the relevant examinations or actively treat other diseases before the operation to reach the standard of surgery, so the interval from injury to preoperation is long, plus long time in bed, higher age and other factors will increase the risk of ICDVT. According to recent studies^[14,15]^ that ICDVT diagnosed by DUS accounted for 30% to 50% of all lower extremity DVTs, therefore, it was speculated that the incidence of ICDVT should theoretically be higher after hip fracture, and it is necessary to identify the associated risk factors so that early interventions can be taken to reduce morbidity, medical costs and patient suffering. However, there is still a lack of data on ICDVT following hip fractures. To our best knowledge, this retrospective, case-control analysis was the first large research focused on the preoperative ICDVT following hip fracture. Despite adherence to chemical thromboprophylaxis and physical anticoagulant measures were adopted from hospitalization to preoperation, but this study had shown that the incidence of ICDVT was still not low, that was 23.5%. It was consistent with the results of previous studies, a related study in Japan^[16]^ showed that ICDVTs accounts for 50% of all cases, many of which were asymptomatic, and were the most common in patients undergoing lower limb fractures surgery, with a rate as high as 60% to 65.3%. An examination of 969 suspected DVT patients in the emergency room in the United Kingdom confirmed that acute ICDVTs accounted for 49.6% of all DVTs.^[17]^ Palareti and Sartori^[18]^ performed intravenous ultrasonography of the whole leg in patients with suspected venous thrombus embolism, and the results showed that the prevalence of ICDVT was 4% to 15% and 7% to 11% in patients with suspected DVT and PE, respectively, while the proportion of patients diagnosed with DVT was 23% to 59%. Another study^[19]^ also believed that if all patients with suspected DVT had received a full-leg intravenous ultrasound examination, the incidence of solitary distal calf DVT should account for half of all DVTs diagnoses. An autopsy observation by Ro and Kageyama^[20]^ based on 100 patients who died of PE showed that 189 lower limbs had calf vein DVT. Among them, nearly 50% of patients had ICDVT, and most of them were fresh thrombus or organic thrombosis. No isolated proximal DVT was found, and the proximal DVTs were mainly fresh thrombus. A similar study^[21]^ was also seen in another application of CT venography to examine the occurrence of DVT in patients with suspected PE. The researchers performed an angiographic examination of 215 patients and found that about 33% had ICDVT, and calf muscular veins were the most common site.

The main risk of ICDVT was that the thrombosis spread to the proximal end to form proximal DVT or the embolus falls off to cause fatal PE. However, recent studies had shown that the risk of DVT or PE secondary to ICDVT might be higher than previous studies. Garry et al^[22]^ summarized the results of 5 randomized clinical trials and 10 prospective cohort studies and found that the rate of ICDVT progressing to the proximal vein can reach 9%, while the rate of PE was close to 1.5%. In a large registration-based study,^[23]^ the incidence of PE in 1885 patients with symptomatic ICDVT within 3 months was 0.7%, of which the rate of potentially fatal PE was 0.3%. In a recent literature report,^[18]^ the proximal extension rate of untreated ICDVT was between 10% and 15%. And there was no reliable evidence that anticoagulant therapy could reduce the incidence of adverse consequences. Righini et al^[24]^ conducted a randomized, doubleblind, placebo-controlled trial involving multiple national medical centres, 122 patients with ICDVT were treated with nadroparin anticoagulation, and 130 patients in the control group were treated with 0.9% normal saline. At the end of follow-up (6 weeks), there was no significant difference in the incidence of proximal DVT or PE between the 2 groups, with the incidence of 3% in the anticoagulation group and 5% in the placebo group. However, the incidence of bleeding was 4% in the anticoagulant group and 0% in the placebo group, therefore, the authors believed that anticoagulant therapy did not reduce the secondary risk of symptomatic DVT, but increased the risk of bleeding. Therefore, the best way to treat ICDVT after hip fracture perhaps was to prevent the onset of the disease, so it was necessary to identify its risk factors to help take measures to reduce the incidence rate. Therefore, we analyzed 44 related factors and eventually found 3 independent risk factors that were closely associated with DVT, including current smoking, time delay from injury to operation, APTT < 28 seconds. The association between current smoking and ICDVT was strengthened with further adjustment for potential confounders in the multivariate logistics regression model, which suggested that smoking was independently associated with ICDVT in hip fracture patients, with a 3.053-fold elevated risk of ICDVT in smokers compared with nonsmokers (95% confidence interval, 1.471-6.335, *P* = .003).

Previous investigations on the relationship between smoking and ICDVT were inconsistently reported and greatly varied,^[25,26]^ while smoking had been shown to act synergistically with other predisposing factors (eg, malignant tumour, older age, cerebrovascular or cardiovascular diseases) in the development of the provoked ICDVT.^[27,28]^ In addition, it had been well-established that cigarette smoking was significantly associated with high plasma fibrinogen levels, leading to prolonged coagulation propensity.^[29,30]^ The riskincreasing impact of smoking might be attributed to multiple pathways or factors it upregulates in the coagulation system, which could be partially explained by the strong relationship between smoking and the presence of ICDVT in patients with hip fractures. Although the explicit connection between cigarette smoking and ICDVT remains unclear, this clinical relevance of the smoking and occurrence of ICDVT should not be ignored, there were other benefits to dissuading inpatients from quitting smoking although smoking was not included in the relevant thrombotic risk assessment score (eg, Caprini score, Padua scores).

Related guidelines^[31,32]^ recommended for patients with a hip fracture should be operated on as soon as possible once their physical condition permitted. It was best to operate within 48 hours at admission. This treatment would be better and could reduce pain and the incidence of complications such as ICDVT. Our study confirmed the factor of time delay from injury to operation was also a significant independent association with preoperative ICDVTs, the reason might be that the later the operation, the longer the patient stayed in bed and the longer the limbs were immobilized, the greater the risk of ICDVT. The related thrombotic risk assessment scores had made it clear that lied in bed and limb immobilization increased the risk of ICDVT. Therefore, patients with hip fractures should be treated with early surgical treatment to reduce the formation of ICDVTs. Our study also showed that the levels of APTT preoperation were closely related to the formation of ICDVT, which were consistent with previous studies,^[33-35]^ indicating that the endogenous coagulation pathway was the main cause of ICDVT in patients with hip fractures. It suggested that patients with hip fracture should pay attention to the change of APTT value in coagulation items. When the value was less than 28 seconds, attention should be paid to the high risk of ICDVT formation, and strict monitoring should be paid to and anticoagulant treatment should be given.

### 4.1. Limitations

Although the data in this study included a large sample, however, there were still some limitations. Firstly, the patients came from 1 single hospital, it only represented the characteristics of 1 region. Second, it was inevitable that there would be recall bias because this retrospective study was dependent on the quality of the data recorded in the medical records. Furthermore, the same to other multivariate analyses, we did not include all relevant risk factors and all patients during observation but on the contrary, we excluded some patients for data deficiency and other patients with several serious comorbidities to improve internal validity, so our conclusions might not be generalizable to these patients. Finally, outpatients were not included in the study, and there may be selection bias.

## 5. Conclusion

In conclusion, the incidence of preoperative ICDVT in hip fracture was 23.5%, and patients with associated risk factors are prone to form ICDVTs, including current smoking, time delay from injury to operation and APTT < 28 seconds. Identification of these factors may help to reduce the incidence of ICDVT by taking early prevention measures.

## Acknowledgments

We acknowledge all the patients who participated in the study and all the staff in the Department of Orthopaedic Surgery.

## Author contributions

**Conceptualization:** Li Liu.

**Data curation:** Tiantian Liu, Li Liu.

**Formal analysis:** Weiguang Zhao, Jianlong Zhao.

**Investigation:** Jianlong Zhao, Tiantian Liu, Li Liu.

**Methodology:** Weiguang Zhao, Jianlong Zhao, Tiantian Liu.

**Resources:** Jianlong Zhao.

**Software:** Jianlong Zhao, Tiantian Liu.

**Supervision:** Zhenwu Liu, Li Liu.

**Writing** - **original draft:** Weiguang Zhao.

**Writing** - **review & editing:** Weiguang Zhao, Zhenwu Liu, Li Liu.
